# Plasma 1α-Hydroxycorticosterone as Biomarker for Acute Stress in Catsharks (*Scyliorhinus canicula*)

**DOI:** 10.3389/fphys.2019.01217

**Published:** 2019-09-20

**Authors:** Ignacio Ruiz-Jarabo, Cristina Barragán-Méndez, Ismael Jerez-Cepa, Miriam Fernández-Castro, Ignacio Sobrino, Juan M. Mancera, Johan Aerts

**Affiliations:** ^1^Faculty of Marine and Environmental Sciences, Department of Biology, Instituto Universitario de Investigación Marina (INMAR), Campus de Excelencia Internacional del Mar (CEI-MAR), Universidad de Cádiz, Cádiz, Spain; ^2^Centro Oceanográfico de Cádiz, Instituto Español de Oceanografía (IEO), Cádiz, Spain; ^3^Stress Physiology Research Group, Faculty of Sciences, Ghent University, Ostend, Belgium; ^4^Stress Physiology Research Group, Animal Sciences Unit, Flanders Research Institute for Agriculture, Fisheries and Food, Ostend, Belgium

**Keywords:** 1α-hydroxycorticosterone, glucocorticoid, *Scyliorhinus canicula*, shark, stress

## Abstract

Glucocorticoids are pleiotropic steroid hormones mediating redistribution of energy. They induce breakdown of glycogen stores and consequent plasma hyperglycaemia after stressful situations. Glucocorticoid actions in most vertebrate species are exerted by cortisol and corticosterone. However, 1α-hydroxycorticosterone is the dominant corticosteroid hormone in elasmobranchs, though its effects as a glucocorticoid are unknown. Here we demonstrate, by using ultra-performance liquid chromatography coupled to tandem mass spectrometry for the quantification of 1α-hydroxycorticosterone in plasma of the elasmobranch *Scyliorhinus canicula*, the response of this hormone to an acute-stress situation and for the first time its glucocorticoid action in elasmobranchs. After an acute air-exposure challenge, *S. canicula* increased plasma levels of 1α-hydroxycorticosterone altogether with enhanced glycolysis and gluconeogenesis pathways to fuel energy demanding tissues, such as white muscle, during the first hours after the stress situation. We foresee our study as a starting point to evaluate stress responses in elasmobranchs, as well as for future applications in the management of these key ecosystem species.

## Introduction

The stress response in vertebrates has been studied in numerous species, whereby an immediate catecholamine mediated response is followed by a hypothalamic-pituitary-interrenal (in fish) or -adrenal (in other vertebrates) axis mediated release of glucocorticoids (Wendelaar Bonga, 1997; Sapolsky et al., 2000). Where catecholamines, in particular (nor)epinephrine, trigger a rapid increase of blood glucose as well as heart rate and blood flow to skeletal muscles (Yaribeygi and Sahraei, 2018), plasma glucocorticoids, in particular cortisol or corticosterone depending on the species, enable the individual to cope with the more sustained energetic demands imposed by the stressor(s) (Wendelaar Bonga, 1997).

Though, the stress response is highly conserved across vertebrates, slight differences were observed between taxa, in particular between fish and other vertebrates (Terrien and Prunet, 2013). In teleost fish, the dominant glucocorticoid cortisol was shown to bind to mineralocorticoid as well as glucocorticoid receptors, hereby also regulating ionic balances. On the contrary, higher vertebrates synthesize the mineralocorticoid aldosterone, which acts as a regulator of ion balance. However, 11-deoxycorticosterone was recently found in fish as potential candidate performing mineralocorticoid functions (Kiilerich et al., 2018).

Most studies on fish were performed on teleost fish, where differences have been described between taxa (Terrien and Prunet, 2013); data are limited for elasmobranchs where 1α-hydroxycorticosterone (1α-OH-B; 1α,11β,21-trihydroxy-4-pregnene-3,20-dione), not cortisol, has been shown to be the dominant hormone (Idler and Kane, 1980). This corticosteroid was first isolated and characterized in 1966 (Idler and Truscott, 1966), and its mineralocorticoid activity has been described (Idler et al., 1967), mostly associated to the loss of plasma urea and sodium retention at lower environmental salinities in lesser spotted catshark (*Scyliorhinus canicula*) (Hazon and Henderson, 1984; Armour et al., 1993). However, to date no data exist for its potential glucocorticoid action in elasmobranchs. Moreover, due to the difficulties in synthesizing this hormone (Anderson, 2012), the analytical methods used in the literature are actually based on other corticosteroids rather than 1α-hydroxycorticosterone (Evans et al., 2010; Brinn et al., 2012), rendering results less physiologically specific.

Taking into account that glucocorticoids are pleiotropic (Barton, 2002), several attempts were made to uncover glucocorticoid actions in elasmobranchs by injecting mammalian adrenocorticotropic hormone (ACTH), cortisol, or even corticosterone in the dogfish (*Squalus acanthias*), however, results on plasma glucose were inconsistent, while glycogen stores where not affected (Patent, 1970; DeRoos and DeRoos, 1973). A potential explanation for these observations was the hypothesis that elasmobranchs may have a reduced reliance on carbohydrates as an energy source (Ballantyne, 1997). However, glycogen levels in the liver of skate (*Dasyatis pastinaca*) were shown to be as high as 2% of the liver weight, while the levels in the muscle were around 0.2% (Leibson and Plisetskaya, 1968), similar to the levels in teleost fish, which rely on glucose mobilization to fuel energy demanding tissues (Vargas-Chacoff et al., 2016). Moreover, plasma glucose variations were described after acute-stress responses in several elasmobranch species (Ellis et al., 2016), highlighting the importance of carbohydrates as energy source in their ability to cope with stress situations.

In addition, lipids were shown as a major source of metabolic energy in sharks (Pethybridge et al., 2014), whereby the liver serves as main lipid storage place and triglycerides (TAG) synthesis site in elasmobranchs (Sargent et al., 1972; Ballantyne, 1997), while fatty acid oxidation in extra-hepatic tissues, such as white muscle, also occurs (Speers-Roesch and Treberg, 2010). However, the literature lacks data on lipid metabolism after short-term stress challenges in elasmobranchs. It was stated that acute stress responses in the teleost fish Senegalese sole (*Solea senegalensis*) included TAG mobilization after air exposure (Costas et al., 2011), but it should be noted that the energy metabolism in elasmobranchs and teleosts is different (Speers-Roesch and Treberg, 2010).

The aim of this study was to evaluate the relation between plasma glucose, lipids, and 1α-hydroxycorticosterone by eliciting an acute stress response in the lesser spotted catshark (*S. canicula*) using a highly accurate and specific ultra-performance liquid chromatography coupled to tandem mass spectrometry (UPLC-MS/MS) quantification method for plasma 1α-hydroxycorticosterone.

## Materials and Methods

### Ethics Statement

This study was performed in accordance with the Guidelines of the European Union (2010/63/UE) and the Spanish legislation (RD 1201/2005 and law 32/2007) for the use of laboratory animals. This study did not involve endangered nor protected species. All experiments have been carried out under a special permit of scientific fishing granted to the Spanish Institute of Oceanography, and approved by the Spanish General Secretariat of Fisheries (project DISCARDLIFE, Fundación Biodiversidad, Ministry for the Ecological Transition, Spain).

### Experimental Procedure

Lesser spotted catshark adults of both sexes (*n* = 46, 380.6 ± 12.0 g body weight and 50.7 ± 0.5 cm total length, mean ± SEM) were obtained by bottom trawling as described before (Barragán-Méndez et al., 2018, 2019) and maintained in the experimental fish facility of the Faculty of Marine and Environmental Sciences (Puerto Real, Cadiz, Spain) for 17 days until the start of the experiment. Fish were randomly divided into six tanks of 400 L (0.72 m^2^ surface area, 0.56 m depth) covered by a fine-mesh tissue for shadowing the aquarium, at a stocking density of 7 or 8 fish per tank, as described for other demersal shark species (Wood et al., 2010; Deck et al., 2016). The system consisted in a flow-through supply of seawater (38 psu), natural photoperiod (November; latitude 36° 31′ 34^″^ N) and temperature (ambient temperature of approximately 19°C) along the acclimation period (17 days). Fish were fed daily at 20.00 h with fresh shrimps, prawns, sardines and anchovies to satiety. Fish were fasted for 36 h before sampling in order to avoid plasma imbalances related to feeding, as described in dogfish (Wood et al., 2007).

Three tanks containing 7 or 8 fish each served as control and were left undisturbed, while fish of three tanks were exposed to air in order to elicit an acute stress response. Hereto, fish were caught by hand and placed in a dry tank for 18 min, being the average time catsharks are exposed to air during commercial fishing procedures (Barragán-Méndez et al., 2019), after which fish were allowed to recover in oxygen-saturated (>90% O_2_ saturation) seawater (all environmental conditions were kept identical to those before the experiment) with fine bubbles from an air stone to ensure maximum gas exchange efficiency. Air-exposure procedures started at 08.30 h. Samples of 2 or 3 fish per tank (total per treatment was 7 or 8 fish) were collected immediately (0 h), 5 and 24 h after air exposure, respectively. As the use of anesthesia was shown to affect stress-related blood variables in sharks (Frick et al., 2009), fish were covered with a wet tissue, while blood from the caudal veins was collected using heparinized syringes and immediately transferred into heparinized tubes. After centrifugation (3 min, 10000 × *g*, 4°C) plasma samples were snap frozen in liquid nitrogen. After blood collection, fish were anesthetized in 0.1% (v/v) 2-phenoxyethanol (P-1126, Sigma-Aldrich) and euthanasia was performed by severing the head with a sharp knife. Sampling lasted less than 4 min per tank, hereby avoiding sampling induced stress. Weight and length of the animals were registered and liver was sampled, weighed, and hepatosomatic index (HSI) was determined. In addition, a part of the liver (from the middle region of the left main lobe) as well as of white muscle (from the upper part of the back situated between the anal fin and the first dorsal fin) were snap frozen in liquid nitrogen. All samples were stored at −80°C until analysis.

### Plasma Glucose and TAG

Plasma glucose and TAG levels were measured using commercially available enzyme immune assays (Glucose-HK ref. 1001200 and TAG ref. 100131101, Spinreact SA, Sant Esteve de Bas, Spain) adjusted for 96-well microplates. All assays were performed on a Bio-Tek PowerWave 340 Microplate spectrophotometer (BioTek Instruments, Winooski, VT, United States) using KCjunior Data Analysis Software.

### Plasma 1α-Hydroxycorticosterone

Taking into account that 1α-hydroxycorticosterone is not commercially available and it has a similar chemical structure and an identical molecular weight as cortisol, two UPLC-MS/MS quantification methods, both developed and validated at the Stress Physiology Research Group (Ghent University, Belgium), for cortisol, its precursors and phase I metabolites (Aerts et al., 2018) and for corticosterone and its phase I metabolites (Salleh Hudin et al., 2017), respectively, were combined and optimized for shark plasma. Subsequently, a wide range of preliminary plasma samples obtained from sharks and rays were analyzed, after which the most abundant ion fragment was used for quantification of plasma 1α-hydroxycorticosterone, while four other ion fragments were chosen for qualification of this hormone.

In brief, after defrosting, the volume of plasma was standardized at 1 mL and pipetted into a 12 mL tube. Subsequently, 3990 μL of water (Type I) and 10 μL of a cortisol-d_4_ of 0.5 μg/L was added as internal standard. When the available plasma volume was less than 1 mL, the volumes of water and internal standard were adjusted accordingly. The mixed solution was vortex-mixed for 30 s to homogenize, ultra-purified using solid phase extraction and analyzed on an Acquity UPLC BEH C18 (1.7 μm; 2.1 mm × 100 mm) column by means of UPLC-MS/MS (Xevo TQS, Waters, Milford, CT, United States). Since in future research matrix-matched calibration curves are not feasible, calibration curves were made in H_2_O/MeOH (80:20, v/v). Subsequently, the stock factor is 100 and results were corrected. Data analysis was performed using Targetlynx software from Waters. Results were reported as the value (ng/mL or μg/L) ± the expanded measurement uncertainty (U) (ng/mL or μg/L) with a coverage factor (k) of 2 (95% confidentiality interval).

### Liver and Muscle Enzymatic Activity and Metabolite Levels

Frozen liver and muscle were finely minced by cutting, homogenized on an ice-cooled petri dish, and divided into two aliquots to assess enzymatic activity and metabolite levels, respectively.

Aliquots for analysis of enzymatic activity were further homogenized by ultrasonic disruption in 10 volumes of ice-cold stop buffer [250 mmol/L sucrose, 50 mmol/L imidazole at pH 7.5, 1 mmol/L 2-mercaptoethanol, 50 mmol/L NaF, 4 mmol/L EDTA, 0.5 mmol/L PMSF, and a protease inhibitor cocktail (Sigma, P-2714)]. The homogenate was centrifuged (10 min, 9000 × *g*, 4°C) after which the supernatant was used for analysis of (i) glycolytic enzymes (glycogen phosphorylase – GP, EC 2.4.1.1; hexokinase – HK, EC 2.7.1.1; pyruvate kinase – PK, EC 2.7.1.40; malate dehydrogenase – MDH, EC 1.1.1.37); (ii) gluconeogenesis-related enzymes (fructose 1,6-bisphosphatase – FBP, EC 3.1.3.11; lactate dehydrogenase – LDH, EC 1.1.1.27); (iii) lipid-related enzymes (glycerol-3-phosphate dehydrogenase – GPDH, EC 1.1.1.8; β-hydroxyacyl-CoA dehydrogenase – HOAD, EC 1.1.1.35); and (iv) phosphate shunt enzyme glucose-6-phosphate dehydrogenase – G6PDH, EC 1.1.1.49), as described for gilthead seabream (*Sparus aurata*) (Laiz-Carrion et al., 2003; Polakof et al., 2006; Vargas-Chacoff et al., 2016) as well as for catsharks and other elasmobranch species (Treberg et al., 2003; Speers-Roesch et al., 2006; Walsh et al., 2006; Deck et al., 2016). MDH and HOAD assays were modified from Niedzwiecka and Skorkowski (2013) and Goertzen et al. (2011), respectively. Conditions for these two enzymes were: MDH was assayed at pH 7.5 with 1 mM MnCl_2_, 0.5 mM NAD^+^, and 5 mM L-malate as substrate; while HOAD activity pH was 7.4 with 0.16 mM NADH and 0.1 mM acetoacetyl CoA as substrate. Activity of these enzymes was normalized to protein concentration in the sample (U mg prot^–1^). Protein was assayed in triplicate for each sample with a BCA Protein Assay Kit (Pierce^TM^, Thermo Fisher Scientific, United States, #23225) using BSA as a standard. Enzymatic activities were determined using a PowerWave^TM^ 340 microplate spectrophotometer (BioTek Instruments, Winooski, VT, United States) using KCjunior Data Analysis Software for Microsoft^®^, Windows XP. Reaction rates of enzymes were determined by changes in absorbance from the reduction of NAD(P)^+^ to NAD(P)H, measured at 340 nm and 37°C, during pre-established times (5–10 min).

Aliquots for analysis of metabolites were further homogenized by ultrasonic disruption in 7.5 volumes ice-cold 0.6 N perchloric acid, neutralized using 1 M potassium bicarbonate, centrifuged (3 min, 10,000 × *g*, 4°C), and the supernatant was used for analysis of: (i) lactate (lactate ref. 1001330, Spinreact SA, Sant Esteve de Bas, Spain); (ii) TAG (see above); (iii) glycogen (Keppler and Decker, 1974); and (iv) glucose obtained after glycogen breakdown (after subtraction of free glucose levels) (see above).

### Statistical Analysis

All dependent variables were normally distributed and their variances were shown homogeneous, by means of the Shapiro-Wilk’s test and the Levene’s test, respectively. Differences between treatments were tested using two-way ANOVA with treatment (control and air exposure) and time (0, 5, and 24 h) as the factors of variance. To achieve normality, data were Log-transformed, where needed. When ANOVA resulted significant differences, a Tukey’s *post hoc* test was used to identify significantly different groups. Correlations between 1α-hydroxycorticosterone relative to all other parameters were analyzed using linear regression on individual values (Ruiz-Jarabo et al., 2017). Statistical significance was accepted at *p* < 0.05. All the results are given as mean ± SEM.

## Results

No mortality occurred during the experiment and all fish showed full physiological recovery within the first 24 h, coinciding with previous studies mimicking fisheries procedures and acute stress situations in other shark species (Cliff and Thurman, 1984; Richards et al., 2003; Kneebone et al., 2013). *P*-values of all parameters assessed in this study are shown in Supplementary File 1.

### Plasma 1α-Hydroxycorticosterone

**FIGURE 1:**
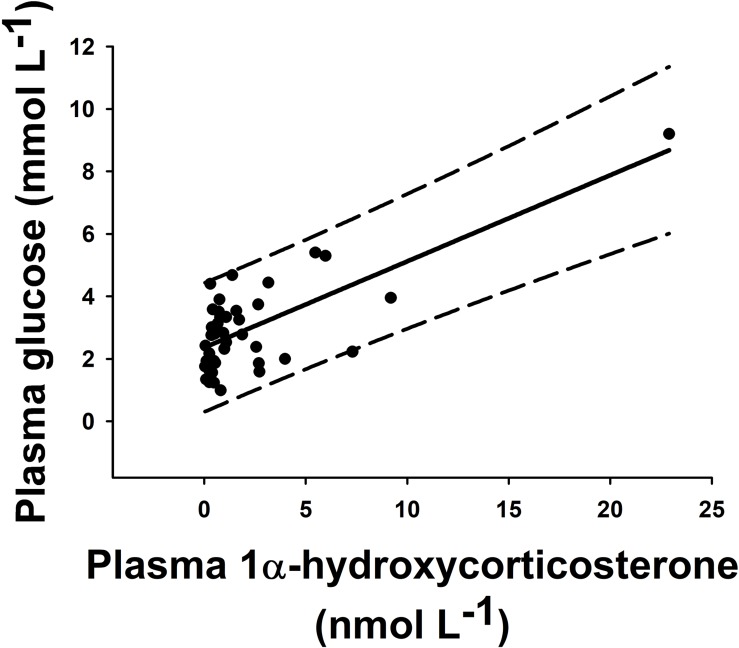
Pairwise correlation of plasma concentration of 1α-hydroxycorticosterone (nmol L^–1^) versus glucose (mmol L^–1^) in individual catsharks (black dots). All animals from this experiment (*n* = 45) were included. These parameters correlated through a linear regression with *r*^2^ = 0.5247 (solid line). Long-dash lines show 95% prediction band.

**FIGURE 2:**
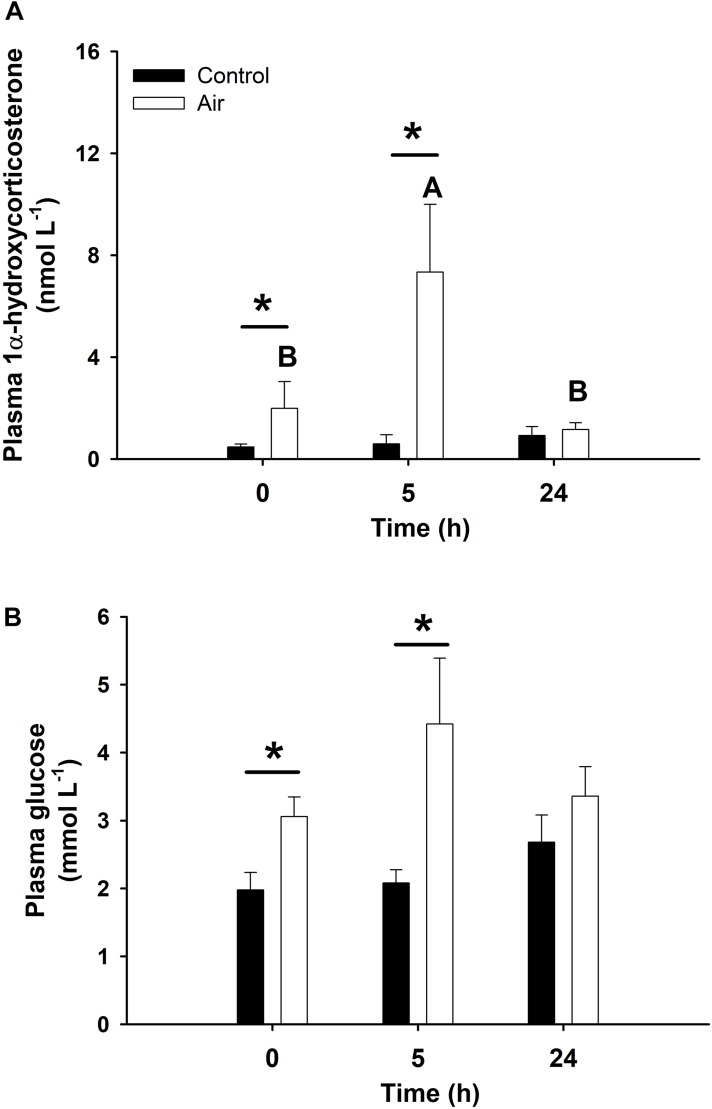
Plasma concentration of 1α-hydroxycorticosterone (**A**, in nmol L^–1^) and glucose (**B**, mmol L^–1^) in catshark after air exposure. Data are expressed as mean ± SEM. Black bars represent control-undisturbed group at each time, while white bars are the air-exposed group. No differences were observed for the control group with time; different capital letters indicate significant differences with time for the air-exposed group; while asterisks (^∗^) indicate significant differences between both groups at each sampling time (*p* < 0.05, two-way ANOVA followed by a Tukey’s *post hoc* test, *n* = 8 for the groups sampled at time 0 h, and for the air-exposed group at 24 h; *n* = 7 for the others).

Statistically significant pairwise correlations between plasma 1α-hydroxycorticosterone and all other parameters are shown in Table 1. Linear correlation analysis revealed that plasma 1α-hydroxycorticosterone only correlated significantly with plasma glucose (Figure 1) and liver lactate (coefficient of determination, *r*^2^, 0.5247 and 0.3960, respectively), while liver free glucose levels correlation was not significant (*r*^2^ = 0.1159). The control group showed no changes in plasma 1α-hydroxycorticosterone at any of the sample points, while the levels in the stressed fish increased significantly after 18 min air exposure (0.47 ± 0.12 and 1.99 ± 1.05 nM for the control and air-exposed groups, respectively), reaching high values 5 h after recovery (7.33 ± 2.67 nM) (Figure 2).

**TABLE 1:** Pairwise product-moment correlation analysis between plasma 1α-hydroxycorticosterone and other parameters analyzed in catsharks after air-exposure for 18 min and subsequent recovery along a time-course including 0, 5, and 24 h.

		**Linear regression**		
**Tissue**	**Parameter**	***r***	***r*^2^**	***p***
Plasma	Glucose	0.724	0.5247	<0.00001
Liver	Glucose	0.340	0.1159	0.0238
	Lactate	0.629	0.3960	<0.00001

*Linear regression results (*n* = 45) are displayed as the Pearson’s coefficient (*r*), coefficient of determination (*r*^2^), and *p*-value (*p*). Only those variables with a significant correlation (*p* < 0.05) are shown.*

### Glycolysis

**FIGURE 3:**
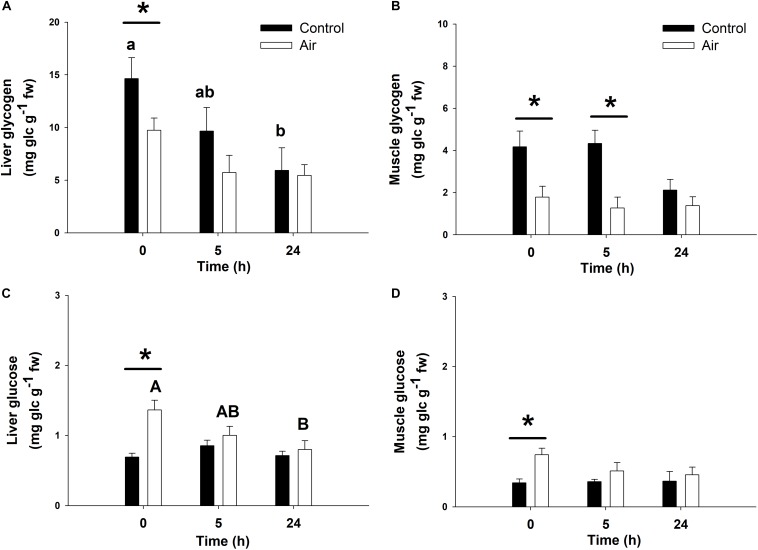
Glycogen and glucose concentrations (mg glucidic units g^–1^ fresh weight) in liver **(A,C)** and muscle **(B,D)** of catshark after air exposure. Data are expressed as mean ± SEM. Black bars represent control-undisturbed group at each time, while white bars are the air-exposed group. Different lowercase and capital letters indicate significant differences with time for the control and air-exposed groups, respectively; while asterisks (^∗^) indicate significant differences between both groups at each sampling time (*p* < 0.05, two-way ANOVA followed by a Tukey’s *post hoc* test, *n* = 8 for the groups sampled at time 0 h, and for the air-exposed group at 24 h; *n* = 7 for the others).

Air exposure induced a glycogen depletion of 33.5% in the liver at 0 h, and of 64.1% at 0 and 5 h in muscle, corresponding to an enhancement of free glucose levels in both tissues at 0 h (Figure 3) and an enhancement of plasma glucose at 0 and 5 h (Figure 2). The activity of GP, which converts glycogen into free glucose, HK, being the first enzyme of the glycolysis pathway, PK, being the last enzyme of the glycolysis pathway, and MDH, an important mediator in the Krebs cycle are shown in Table 2. High levels of free glucose in liver coincided with an increase in hepatic GP activity at 0 h in the air-exposed group (Table 2). HK decreased significantly in hepatic tissue at 0 h in the air-exposed group, while increased significantly in liver and white muscle at 5 h. PK activity decreased significantly in liver at 0 h, and muscle at 5 h. No changes were observed in MDH activity.

**TABLE 2:** Changes in glycolytic-related enzymes (glycogen phosphatase, GP; hexokinase, HK; pyruvate kinase, PK; and malate dehydrogenase, MDH) activities (U mg prot^–1^) in liver and muscle of catshark after air exposure.

**Tissue**	**Enzyme**	**Group**	**0 h**	**5 h**	**24 h**
Liver	GP	Control	0.47 ± 0.05	0.47 ± 0.05	0.48 ± 0.04
		Air	0.71 ± 0.05 A^∗^	0.47 ± 0.03 B	0.40 ± 0.05 B
Liver	HK	Control	0.10 ± 0.01	0.12 ± 0.01	0.09 ± 0.02
		Air	0.05 ± 0.01 C^∗^	0.16 ± 0.02 A^∗^	0.10 ± 0.01 B
Liver	PK	Control	7.2 ± 0.7 b	12.0 ± 1.0 a	9.9 ± 0.5 ab
		Air	8.4 ± 0.9	9.2 ± 0.8^∗^	8.7 ± 0.7
Liver	MDH	Control	0.06 ± 0.01	0.06 ± 0.02	0.08 ± 0.02
		Air	0.06 ± 0.01	0.05 ± 0.01	0.08 ± 0.01
Muscle	GP	Control	0.31 ± 0.14	0.18 ± 0.08	0.60 ± 0.28
		Air	0.45 ± 0.37	0.19 ± 0.10	0.33 ± 0.08
Muscle	HK	Control	0.21 ± 0.03	0.21 ± 0.05	0.16 ± 0.06
		Air	0.07 ± 0.02 B	0.43 ± 0.09 A^∗^	0.20 ± 0.06 B
Muscle	PK	Control	1.9 ± 0.4 a	0.8 ± 0.2 b	1.1 ± 0.1 ab
		Air	0.6 ± 0.1^∗^	0.8 ± 0.1	1.1 ± 0.2
Muscle	MDH	Control	0.06 ± 0.02	0.07 ± 0.03	0.14 ± 0.02
		Air	0.07 ± 0.02	0.07 ± 0.03	0.07 ± 0.03

*Data are expressed as mean ± SEM. Different lowercase and capital letters indicate significant differences with time for the control and air-exposed groups, respectively; while asterisks (^∗^) indicate significant differences between both groups at each sampling time (*p* < 0.05, two-way ANOVA followed by a Tukey’s *post hoc* test, *n* = 8 for the groups sampled at time 0 h, and for the air-exposed group at 24 h; *n* = 7 for the others).*

### Anaerobic Metabolism and Gluconeogenesis

**FIGURE 4:**
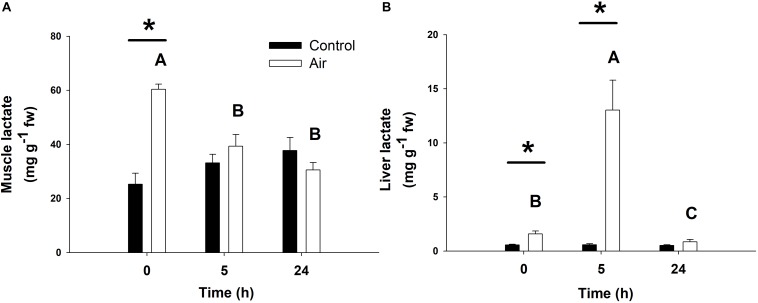
Lactate concentrations (in mg lactate g^–1^ fresh weight) in muscle **(A)** and liver **(B)** of catshark after air exposure. Data are expressed as mean ± SEM. Black bars represent control-undisturbed group at each time, while white bars are the air-exposed group. No differences were observed for the control group with time; different capital letters indicate significant differences with time for the air-exposed group; while asterisks (^∗^) indicate significant differences between both groups at each sampling time (*p* < 0.05, two-way ANOVA followed by a Tukey’s *post hoc* test, *n* = 8 for the groups sampled at time 0 h, and for the air-exposed group at 24 h; *n* = 7 for the others).

The stress response following air-exposure elicited an immediate increase in muscle lactate levels at 0 h, and in liver at 0 and 5 h (Figure 4). An increase in plasma lactate levels after air-exposure was previously observed in a complementary study (Barragán-Méndez et al., 2019). Gluconeogenic enzymes such as LDH, which converts lactate into pyruvic acid, and FBP, which converts 1,6-biphosphate to fructose 6-phosphate in gluconeogenesis, are shown in Table 3. Hepatic LDH activity increased significantly at 5 h in the air-exposed group, while no changes occurred in white muscle at any of the sampling time points. Hepatic FBP activity in muscle decreased in the stressed fish at 5 h, while increased at 0 h.

**TABLE 3:** Changes in gluconeogenic-related enzymes (lactate dehydrogenase, LDH; and fructose 1,6-bisphosphatase, FBP) activities (U mg prot^–1^) in liver and muscle of *S. canicula* after air exposure.

**Tissue**	**Enzyme**	**Group**	**0 h**	**5 h**	**24 h**
Liver	LDH	Control	0.07 ± 0.01	0.05 ± 0.01	0.10 ± 0.01
		Air	0.07 ± 0.01	0.11 ± 0.02^∗^	0.08 ± 0.02
Liver	FBP	Control	1.11 ± 0.02	1.14 ± 0.05	1.15 ± 0.05
		Air	1.04 ± 0.04	1.01 ± 0.04^∗^	1.09 ± 0.04
Muscle	LDH	Control	0.08 ± 0.03	0.05 ± 0.02	0.10 ± 0.02
		Air	0.08 ± 0.02	0.03 ± 0.01	0.08 ± 0.02
Muscle	FBP	Control	0.08 ± 0.03	0.09 ± 0.02	0.16 ± 0.04
		Air	0.39 ± 0.07 A^∗^	0.11 ± 0.02 B	0.10 ± 0.03 B

*Data are expressed as mean ± SEM. Different capital letters indicate significant differences with time for the air-exposed groups; while asterisks (^∗^) indicate significant differences between both groups at each sampling time (*p* < 0.05, two-way ANOVA followed by a Tukey’s *post hoc* test, *n* = 8 for the groups sampled at time 0 h, and for the air-exposed group at 24 h; *n* = 7 for the others).*

### Lipid Metabolism

No major changes were observed regarding lipid metabolism. However, an increase in muscle TAG was observed at 0 h in the air-exposed group, followed by a decrease in plasma TAG at 5 h in this group (Table 4). The activity of G6PDH, which is involved in the pentose shunt, GPDH, which serves as a major link between carbohydrate and lipid metabolism, and HOAD, which participates in the beta oxidation of fatty acids in liver and muscle, are shown in Table 5. Muscle tissue of air-exposed fish increased in G6PDH activity at 5 h compared to the control group. Hepatic G6PDH activity decreased significantly at 0 and 5 h when compared to the control group. In liver, G6PDH activity decreased in the air-exposed group at 5 h compared to the control group. No changes were observed for GPDH in muscle nor for HOAD activity in liver as well as in white muscle through-out the experiment.

**TABLE 4:** Changes in triglycerides (TAG) in liver and muscle (mg g^–1^ fresh weight) and plasma (mmol L^–1^) of catshark after air exposure.

**Tissue**	**Group**	**0 h**	**5 h**	**24 h**
Liver	Control	19.7 ± 2.0	17.2 ± 2.4	22.7 ± 1.7
	Air	20.5 ± 2.3	20.0 ± 3.0	24.1 ± 4.5
Muscle	Control	0.84 ± 0.07	0.95 ± 0.04	0.92 ± 0.09
	Air	1.13 ± 0.07^A*^	0.83 ± 0.11B	0.93 ± 0.04B
Plasma	Control	1.97 ± 0.20	2.06 ± 0.24	1.96 ± 0.08
	Air	1.73 ± 0.16	1.29 ± 0.23^∗^	1.50 ± 0.16

*Data are expressed as mean ± SEM. Different capital letters indicate significant differences with time for the air-exposed groups; while asterisks (^∗^) indicate significant differences between both groups at each sampling time (*p* < 0.05, two-way ANOVA followed by a Tukey’s *post hoc* test, *n* = 8 for the groups sampled at time 0 h, and for the air-exposed group at 24 h; *n* = 7 for the others).*

**TABLE 5:** Changes in lipid metabolism-related enzymes (glucose-6-phosphate dehydrogenase, G6PDH; glycerol-3-phosphate dehydrogenase, GPDH; and β-hydroxyacyl coenzyme A dehydrogenase, HOAD) activities (U mg prot^–1^) in liver and muscle of catshark after air exposure.

**Tissue**	**Enzyme**	**Group**	**0 h**	**5 h**	**24 h**
Liver	G6PDH	Control	0.71 ± 0.06 b	0.98 ± 0.07 a	0.79 ± 0.10 ab
		Air	0.47 ± 0.04 B^∗^	0.71 ± 0.13 A^∗^	0.77 ± 0.06 A
Liver	GPDH	Control	1.87 ± 0.08	2.11 ± 0.07	1.92 ± 0.09
		Air	1.99 ± 0.07	1.73 ± 0.10^∗^	1.87 ± 0.06
Liver	HOAD	Control	7.4 ± 0.5	7.5 ± 0.7	7.1 ± 0.5
		Air	8.7 ± 0.6	7.1 ± 0.7	8.7 ± 0.7
Muscle	G6PDH	Control	0.01 ± 0.00 a	0.00 ± 0.00 b	0.01 ± 0.00 a
		Air	0.01 ± 0.00	0.01 ± 0.00^∗^	0.01 ± 0.00
Muscle	GPDH	Control	0.06 ± 0.02	0.08 ± 0.02	0.13 ± 0.04
		Air	0.04 ± 0.01	0.05 ± 0.02	0.09 ± 0.04
Muscle	HOAD	Control	0.18 ± 0.04	0.14 ± 0.03	0.10 ± 0.02
		Air	0.10 ± 0.02	0.13 ± 0.03	0.13 ± 0.02

*Data are expressed as mean ± SEM. Different lowercase and capital letters indicate significant differences with time for the control and air-exposed groups, respectively; while asterisks (^∗^) indicate significant differences between both groups at each sampling time (*p* < 0.05, two-way ANOVA followed by a Tukey’s *post hoc* test, *n* = 8 for the groups sampled at time 0 h, and for the air-exposed group at 24 h; *n* = 7 for the others).*

## Discussion

Overall, our results show that after an acute air-exposure challenge, catsharks increased their plasma 1α-hydroxycorticosterone levels, together with enhanced glycolysis and gluconeogenesis, in order to fuel glucose levels in energy demanding tissues such as white muscle during the first hours after a stress situation.

Plasma 1α-hydroxycorticosterone was quantified using UPLC-MS/MS providing the needed accuracy and specificity as it was based on validated methods (Aerts et al., 2018; Salleh Hudin et al., 2018). The concentrations of 1α-hydroxycorticosterone in our study ranged between 0.01 and 0.83 μg/100 mL plasma (0.04–22.89 nM, including all control and air-exposed fish along the experimental period), and were found to be in the range previously described in the literature for this species (0.36 ± 0.1 μg/100 mL) as well as other species of shark such as spiny dogfish (*S. acanthias*) (2.3 ± 0.5 μg/100 mL), blue shark (*Prionace glauca*) (0.87 ± 0.05 μg/100 mL), shortfin mako (*Isurus oxyrinchus*) (5.3 μg/100 mL) and several other rays and skates species (0.08–4.7 μg/100 mL) measured by a wide range of techniques such as radioimmunoassay, thin layer chromatography coupled to fluorescence or double isotope derivative assay (Anderson, 2012).

Our results provide the needed data for 1α-hydroxycorticosterone glucocorticoid actions in sharks as a significant positive correlation between plasma 1α-hydroxycorticosterone and plasma glucose after an acute stressor was observed, whereby levels of both parameters increased minutes after the air-exposure challenge and reached their highest recorded level 5 h later. A similar response was also described in air-exposed teleost fishes, where plasma cortisol significantly increased 15 min after the start of the exposure, with a maximum concentration 30–60 min later, while plasma glucose increased gradually after the stressor with maximum levels a few hours later (Costas et al., 2011; Skrzynska et al., 2018). As we did not sample our fish between the start of the recovery period (i.e., after 18 min air exposure) and 5 h later, we cannot pinpoint the time point at which the maximum concentration of both parameters occurs. Our findings are further supported as previous studies have shown that increasing plasma ACTH induced a significant increase in cortisol synthesis in teleost fish (Flik et al., 2006) or 1α-hydroxycorticosterone synthesis in catshark (Hazon and Henderson, 1984), respectively.

When focusing on carbohydrate metabolism, glycogen breakdown following an acute stressor was supported as GP activity in the liver increased while glycogen stores in liver, as well as in muscle, decreased indicating the production of glucose. It should be mentioned that muscle GP activity showed no changes along the time in any of the experimental groups. This appreciable lack of response may be due to multiple reasons, including the low activity of this enzyme in this tissue, or limitations of the methodology employed (unable to discern changes as small as there may be in the muscle). However, both liver and muscle GP increased their activities ∼50% at time 0 h in the air-exposed group compared to the control undisturbed group (though no statistical differences are described in muscle). As GP is not regulated by plasma catecholamines in elasmobranchs (Ballantyne, 1997), we can assume the observed enhanced activity was due to other factors such as 1α-hydroxycorticosterone. This reasoning is further supported as corticosterone was shown to increase glycogenolysis in rat (*Rattus norvegicus* domestica) hepatocytes (Gomez-Munoz et al., 1989). In addition, free glucose levels were increased at 0 and 5 h after the onset of the stressor in liver (the main glycogen store in elasmobranchs) and muscle (Leibson and Plisetskaya, 1968) and were even further supported by the increased HK, being the first enzyme in the glycolysis pathway, activity.

In all, these data provide evidence for an activated glycolytic pathway and subsequent increased plasma glucose levels after an acute stressor such as air exposure as seen in our study, but also after capture and/or transport (Cliff and Thurman, 1984; Hoffmayer and Parsons, 2001; Skomal and Mandelman, 2012). However, other studies on elasmobranchs and chondrichthyan species reported lower or even no changes in plasma glucose levels coinciding with higher plasma lactate after an acute stress situation (Manire and Hueter, 2001; Brill et al., 2008; Kneebone et al., 2013; Martins et al., 2018). If glycogenolysis and gluconeogenesis cannot match the rise in anaerobic metabolism, this would lead to the hypoglycaemia observed in previous studies. Our results demonstrated increased burst performance and increased glycolytic processes in peripheral tissues fuelled by the high levels of glucose described in liver, muscle and plasma immediately after the stressor(s). In this sense, enhanced anaerobic glycolysis resulted in a 2.4-fold increase in muscle lactate after air exposure at 0 h. Decreased PK activity in liver and muscle at 0 and 5 h, respectively, further supports this line of reasoning, as PK is the last enzyme in the glycolysis pathway oxidizing phosphoenolpyruvate into pyruvate, which is hereby converted into lactate anaerobically confirming studies on glycogen depletion and accumulation of lactate in white muscle in fish (Wood, 1991), and increased plasma lactate levels in elasmobranchs after exhaustive exercise stress (Frick et al., 2010a, b, 2012). Plasma lactate levels after air exposure paralleled those in the muscle, returning to basal-control levels 5 h after the onset of the acute stressor (Barragán-Méndez et al., 2019). It was described that gluconeogenesis is only performed in the liver (Moon and Mommsen, 1987), which coincides with the observed increased lactate levels in liver after 5 h and the increased activity of LDH and FBP, respectively, converting lactate and other non-carbohydrate molecules into glucose (Suarez and Mommsen, 1987). Furthermore, our data also show increased FBP activity in white muscle immediately after air exposure, indicating the potential presence of gluconeogenic processes in this tissue, although further studies are needed to substantiate this.

When focusing on lipid metabolism, our results indicated that lipid metabolism was not significantly modified after the acute stressor, as no changes were observed in the activity of HOAD nor GPDH in liver and muscle. However, muscle TAG levels increased immediately after air exposure, which supports the finding that catabolism of lipids release glycerol for gluconeogenesis in the liver, but not in muscle of elasmobranchs due to the absence of glycerol kinase in this tissue (Ballantyne, 1997). Interestingly, the increased muscle TAG may be somehow related to the increased FBP activity in this tissue, hereby further supporting the hypothesis that gluconeogenesis occurs in muscle of elasmobranchs. However, further studies are needed to confirm this and to describe the specific pathways that may occur in this tissue.

## Conclusion

We show that after an acute air-exposure challenge, catsharks increased their plasma 1α-hydroxycorticosterone levels altogether with enhanced glycolysis and gluconeogenesis in order to fuel glucose levels in energy demanding tissues such as white muscle during the first hours after a stress situation. This study hereby contributes to the quantification and further understanding of stress in elasmobranchs pivotal in ecology and fisheries management and conservation of these species.

## Data Availability Statement

All datasets generated for this study are included in the manuscript/Supplementary Files.

## Ethics Statement

This study was performed in accordance with the Guidelines of the European Union (2010/63/UE) and the Spanish legislation (RD 1201/2005 and law 32/2007) for the use of laboratory animals. This study did not involve endangered nor protected species. All experiments have been carried out under a special permit of scientific fishing granted to the Spanish Institute of Oceanography, and approved by the Spanish General Secretariat of Fisheries (project DISCARDLIFE, Fundación Biodiversidad, Ministry for the Ecological Transition, Spain).

## Author Contributions

IR-J, CB-M, IS, and JM conceived and designed the experimental study. JA conceived and developed the UPLC-MS/MS method for quantification of plasma 1α-hydroxycorticosterone, and analyzed the plasma for 1α-hydroxycorticosterone. IR-J, CB-M, IJ-C, and MF-C carried out the experimental procedures. IR-J, CB-M, IJ-C, MF-C, and JA analyzed and interpreted the data. IR-J, CB-M, IS, JM, and JA wrote the manuscript. All authors reviewed, edited, and approved the final manuscript.

## Conflict of Interest

The authors declare that the research was conducted in the absence of any commercial or financial relationships that could be construed as a potential conflict of interest.
