# Supplementary material for: Plasma 1α-Hydroxycorticosterone as Biomarker for Acute Stress in Catsharks (Scyliorhinus canicula)
Source: Front Physiol. 2019 Sep 20;10:1217. doi: 10.3389/fphys.2019.01217 (PMC6764463; doi:10.3389/fphys.2019.01217)
Supplement: Supplementary file 1 [file Table_1.docx]

**Ruiz-Jarabo *et al.***

**Supplementary file 1.** P-values from two-way ANOVA of parameters measured in plasma, liver, and muscle of catshark in a time-course experiment following air exposure. Group (control and air-exposed) and time (0, 5 and 24 h) are the main factors. *NS* not significant (*p* > 0.05).

| **Tissue** | **Parameter** | **Group** | **Time** | **Group*Time** |
| --- | --- | --- | --- | --- |
| **Plasma** | 1α-OH-B | <0.00001 | *NS* | <0.005 |
|  | Glucose | <0.001 | *NS* | *NS* |
|  | TAG | <0.005 | *NS* | *NS* |
| **Liver** | Glycogen | <0.05 | <0.002 | *NS* |
|  | Glucose | <0.002 | <0.05 | <0.02 |
|  | Lactate | <0.000001 | <0.000001 | <0.000001 |
|  | TAG | *NS* | *NS* | *NS* |
|  | GP | *NS* | <0.01 | <0.005 |
|  | HK | *NS* | <0.0005 | <0.01 |
|  | PK | *NS* | <0.005 | <0.05 |
|  | MDH | *NS* | *NS* | *NS* |
|  | LDH | *NS* | *NS* | <0.02 |
|  | FBP | <0.02 | *NS* | *NS* |
|  | G6PDH | <0.01 | <0.005 | NS |
|  | GPDH | *NS* | *NS* | <0.02 |
|  | HOAD | *NS* | *NS* | *NS* |
| **Muscle** | Glycogen | <0.0001 | 0.074 | *NS* |
|  | Glucose | <0.01 | *NS* | *NS* |
|  | Lactate | <0.0005 | <0.05 | <0.00001 |
|  | TAG | *NS* | *NS* | <0.05 |
|  | GP | *NS* | *NS* | *NS* |
|  | HK | *NS* | <0.005 | <0.01 |
|  | PK | <0.03 | *NS* | <0.02 |
|  | MDH | *NS* | *NS* | *NS* |
|  | LDH | *NS* | 0.053 | *NS* |
|  | FBP | <0.05 | *NS* | <0.02 |
|  | G6PDH | *NS* | *NS* | <0.01 |
|  | GPDH | *NS* | *NS* | *NS* |
|  | HOAD | *NS* | *NS* | *NS* |
